# News and Coming Events

**Published:** 1909-08-07

**Authors:** 


### News and Coming Events.

Lord Sandhurst, Treasurer of St. Bartholomew's Hospital, has received from the Worshipful Company of Skinners the sum of £200, being the first instalment of a grant of £1,000 towards the capital fund of the hospital.

The Vice-Chancellor of London University, Professor M. H. M. Hill, Sc.D., F.R.S., has appointed Dr. A. D. Waller, M.D., C.M., LL.D., F.R.S., Director of the Physiological Laboratory, to represent the University at the inauguration of Dr. A. L. Lowell as President of Harvard College on October 6 and 7.

To provide for larger demands upon the resources of the University next session, the Senate of Belfast University has sanctioned the expenditure of £1,500 for the better equipment of the departments of physiology and pathology, pathological laboratory, physics, chemistry, zoology, archæology, anatomy, botany, and geology.

Dr. William Wilson, M.R.C.S., L.R.C.P., secretary since 1901 of the Friends' Foreign Mission Association, died at Hitchin on July 27 from heart failure, following an operation for appendicitis. Dr. Wilson went out as a missionary to Madagascar in 1877, and in 1880 he came home for medical training, and subsequently qualified. At the time of the French occupation Dr. Wilson was in charge of the Friends' Hospital at Tananarive. His loss will be felt deeply in the missionary world.

The following Fellows of the Royal College of Physicians of London have been elected officers for the ensuing collegiate year :—Censors : Sir W. Allchin, M.D., F. de Havilland Hall, M.D., Seymour J. Sharkey, M.D., J. Kingston Fowler, M.D. Treasurer : Sir Dyce Duckworth, M.D., LL.D. Emeritus Registrar : Dr. Edward Liveing. Registrar : J. A. Ormerod, M.D. Harveian Librarian : Dr. J. Frank Payne. Members of the Library Committee : Norman Moore, M.D., W. Osler, M.D., F.R.S., A. F. Voelcker, M.D., C. A. Mercier, M.D. Curators of the Museum : Sir William Allchin, M.D., S. J. Sharkey, M.D., W. Hunter, M.D., and F. W. Andrewes, M.D.

### BRITISH MEDICAL ASSOCIATION.

The concluding day's proceedings of the British Medical Association were entered upon on July 30 in the Queen's University, Belfast. Several of the sectional meetings were attended by the Lord-Lieutenant and the Countess of Aberdeen. The concluding business meeting was held in the library, when, in the presence of the Viceregal party, Sir William Whitla (president), and a very large number of the members, Sir John W. Byers, M.D., Belfast, delivered an address on the present position and the future developments of obstetric medicine. In reply to a vote of thanks to the Lord-Lieutenant and the Countess of Aberdeen, proposed by Sir William Whitla, Lord Aberdeen said he ventured to say that one of the dominant features of the week's addresses was the brightness and hopefulness that seemed to permeate them, and which would help and stimulate not only the members of their great profession, but that larger public who were paying attention to what was taking place at their meetings. They would all be delighted when the association came again to Ireland, but he hoped it would meantime visit Aberdeen, where they would have a warm welcome. The Viceregal party in the evening attended a reception given to the members of the association by the president and members of the Ulster branch.

The King has been graciously pleased to sanction the appointment as a Knight of Grace in the Order of the Hospital of St. John of Jerusalem in England of Mr. Fred Hibbert Westmacott, F.R.C.S., who has previously been an Honorary Associate of the Order.

The Baly medal, founded by Dr. F. D. Dyster in 1866 "*in memoriam* Gulielmi Baly, M.D.," and awarded every alternate year, on the recommendation of the President and Council of the Royal College of Physicians of London, to the person who shall be deemed to have most distinguished himself in the science of physiology, has been awarded to Emil Fischer, Ph.D., Professor of Chemistry in the University of Berlin; and the Moxon medal, founded in 1890, in memory of Walter Moxon, M.D., and awarded every third year to the person who should be deemed to have most distinguished himself by observation and research in clinical medicine, to Sir William R. Gowers, M.D., F.R.S.

A report by Professor Karl Pearson was presented at a recent meeting of the Senate of London University, showing the progress of the work of the Francis Galton Laboratory for National Eugenics during the past 16 months. The Senate have voted their cordial thanks to Sir Francis Galton for a further donation of £500 for the maintenance of the laboratory, and recorded their high appreciation of the services rendered by Professor Pearson, under whose supervision the work is carried on. Mr. David Heron and Miss E. M. Elderton have been reappointed respectively Galton Research Fellow and Galton Research Scholar for a year from next February.

### PUBLIC SLAUGHTER-HOUSES.

The following Memorial has been presented by the Humanitarian League to the Right Hon. John Burns, M.P., President of the Local Government Board :—

Sir,—In view of the fact that it is over four years since the Committee appointed by the Admiralty to consider the Humane Slaughtering of Animals reported strongly in favour of replacing private slaughter-houses by public abattoirs; that the same course was advocated in the Report of the Royal Commission on Tuberculosis in 1896, and has been urgently recommended by the Public Health Committee of the London County Council and by a number of leading medical and sanitary authorities; and that London is in this respect far behind the great majority of continental cities, and not a few provincial ones, such as Glasgow and Edinburgh; we desire to appeal to you to take the necessary steps to give effect, in the Metropolitan district, to this much-needed but long-delayed reform.

Among the signatories are the following eminent members of the medical profession :—

Sir James Crichton-Browne, M.D., D.Sc., LL.D., F.R.S.
Sir Thomas Barlow, Bart., M.D., D.Sc., F.R.C.P., etc.
Sir Samuel Wilks, Bart., M.D., LL.D., F.R.S., etc. (Consulting Physician to Guy's Hospital).
Sir Jonathan Hutchinson, F.R.S., F.R.C.S.
J. Mitchell Bruce, Esq., M.A., LL.D., M.D., F.R.C.P.
James Cantlie, Esq., M.B., F.R.C.S.
Alex. Hill, Esq., M.A., M.D., F.R.C.S., J.P.
Alex. Haig, Esq., M.A., M.D.
Sir Arthur Conan Doyle, M.D.
The Medical Officers of Health for Bermondsey, Deptford, Fulham, Greenwich, Hackney, Hampstead, Holborn, Islington, Lambeth, St. Marylebone, Shoreditch, Southwark, Stepney, Stoke Newington, and Woolwich.

July 29, 1909.

Miss Ivy E. Woodward, M.D.London, having passed the required examinations, was admitted, at the last comitia of the Council of the College, a member of the Royal College of Physicians of London. Dr. Woodward is thus the first female student admitted to the membership of the College. She received her medical education at the London School of Medicine for Women, in connection with the Royal Free Hospital, and she has held the posts of clinical assistant, house physician, and assistant clinical pathologist. She is also clinical assistant at the Royal Hospital for Diseases of the Chest and the New Hospital for Women.

Among recent questions and answers printed and circulated with the papers before the House of Commons, the following referred to the status of women in the Royal College of Surgeons. Dr. Rutherford asked the Home Secretary whether, in view of the fact that the Royal College of Surgeons of England was unable, through the provisions of the Medical Act of 1876, to confer the rights and privileges of the college to women who were admitted to its dipomas, he proposed to grant a new charter to the college; to which Mr. Gladstone replied : I have no power to take any such action. I understand that a supplementary charter for the Royal College could only be granted by the Crown on the petition of the governing body.

The annual meeting of members of the Association for Promoting the Training and Supply of Midwives was held recently at Chelsea. Mrs. Wallace Bruce, who presided, said that a great change had taken place since the passing of the Midwives Act in 1902. Until then two-thirds of births in the country took place without trained supervision, but now this proportion is about to be reversed. Last year the association trained 20 nurses; this year they were training 40. Next year the Act would come finally into operation; no untrained woman would be able to practise, and the need for trained women would be great. All the funds now at their disposal would be exhausted that year, and they would have to make a fresh appeal to public generosity.

### THE METROPOLITAN ASYLUMS BOARD IN 1908.

The annual report of the Metropolitan Asylums Board for 1908 came before the managers on Saturday, July 17, at a meeting presided over by Mr. J. T. Helby, the Chairman. The report, which was submitted by the Statistical Committee and adopted, stated that although the large number of fever and diphtheria patients under treatment in the board's hospitals during the spring of the year caused some apprehension, the maximum number under treatment (reached on November 30) was 1,856 below the highest level reached in 1907, which was a record year, as many as 7,158 cases having been at one time under treatment. The total number of patients admitted during 1908 was 27,967, with 1,255 deaths. The mortality per cent. was : Scarlet fever, 2.56; diphtheria, 9.73; enteric, 16.28; cerebro-spinal meningitis, 40.0; other diseases, 5.68. Not a single case of smallpox was admitted into the board's hospitals from the Metropolis, though one case was received from Leyton. The cases of mistaken diagnosis in respect of infectious diseases other than smallpox which were admitted last year numbered 2,594, the percentage of error among cases certified as scarlet fever being 6.1, among diphtheria cases 22.2, and among enteric fever cases 39.1. Owing to the death of Dr. Hume, medical superintendent of the North-Western Hospital, the Hospitals Committee recommended, and it was resolved, subject to the assent of the Local Government Board, that Dr. J. McCombie be transferred from the medical superintendentship of the Brook Hospital to that of the North-Western Hospital. Dr. McCombie is the senior medical superintendent under the board, and he has intimated his willingness to be transferred.

At Swansea Assizes on Saturday last, Dr. Howell Thomas Evans, a medical practitioner at Blackwood, Monmouth, claimed damages from the Great Western Railway for personal injuries, and was awarded £3,500 damages and costs. The plaintiff's train, on a journey from Cardiff to Newport, collided with a mineral train, and one coach telescoped another. Dr. Evans and the other occupants of his compartment had to be rescued through the window.

The Royal Boscombe Hospital, Bournemouth, is to be congratulated on an anonymous gift of £4,000 to defray the cost of erecting a ward for children. The new block will include a large ward containing 12 cots, with a cubic air-space of 1,462 feet per patient, and a floor area of 112 feet. The new ward will have a large covered balcony facing south, and will contain in addition bathrooms, ward-kitchen, larder and surgical-store, together with rooms for patients' clothes and linen. Care is to be taken to make the new block as hygienically perfect as possible.

